# Protection of hybrid immunity against SARS-CoV-2 reinfection and severe COVID-19 during periods of Omicron variant predominance in Mexico

**DOI:** 10.3389/fpubh.2023.1146059

**Published:** 2023-04-04

**Authors:** José Antonio Montes-González, Christian Arturo Zaragoza-Jiménez, Neftali Eduardo Antonio-Villa, Carlos A. Fermín-Martínez, Daniel Ramírez-García, Arsenio Vargas-Vázquez, Rosaura Idania Gutiérrez-Vargas, Gabriel García-Rodríguez, Hugo López-Gatell, Sergio Iván Valdés-Ferrer, Omar Yaxmehen Bello-Chavolla

**Affiliations:** ^1^Dirección General de Epidemiología, Secretaría de Salud, Mexico City, Mexico; ^2^Department of Endocrinology, Instituto Nacional de Cardiología Ignacio Chávez, Mexico City, Mexico; ^3^Dirección de Investigación, Instituto Nacional de Geriatría, Mexico City, Mexico; ^4^MD/PhD (PECEM) Program, Facultad de Medicina, Universidad Nacional Autónoma de Mexico, Mexico City, Mexico; ^5^Subsecretaría de Prevención y Promoción de la Salud, Secretaría de Salud, Mexico City, Mexico; ^6^Departamento de Neurología, Instituto Nacional de Ciencias Médicas y Nutrición Salvador Zubirán, Mexico City, Mexico

**Keywords:** reinfection, hybrid immunity, vaccination, vaccine boosters, SARS-CoV-2, heterologous booster, omicron variant (B.1.1.529)

## Abstract

**Background:**

With the widespread transmission of the Omicron SARS-CoV-2 variant, reinfections have become increasingly common. Here, we explored the role of immunity, primary infection severity, and variant predominance in the risk of reinfection and severe COVID-19 during Omicron predominance in Mexico.

**Methods:**

We analyzed reinfections in Mexico in individuals with a primary infection separated by at least 90 days from reinfection using a national surveillance registry of SARS-CoV-2 cases from March 3rd, 2020, to August 13th, 2022. Immunity-generating events included primary infection, partial or complete vaccination, and booster vaccines. Reinfections were matched by age and sex with controls with primary SARS-CoV-2 infection and negative RT-PCR or antigen test at least 90 days after primary infection to explore reinfection and severe disease risk factors. We also compared the protective efficacy of heterologous and homologous vaccine boosters against reinfection.

**Results:**

We detected 231,202 SARS-CoV-2 reinfections in Mexico, most occurring in unvaccinated individuals (41.55%). Over 207,623 reinfections occurred during periods of Omicron (89.8%), BA.1 (36.74%), and BA.5 (33.67%) subvariant predominance and a case-fatality rate of 0.22%. Vaccination protected against reinfection, without significant influence of the order of immunity-generating events and provided >90% protection against severe reinfections. Heterologous booster schedules were associated with ~11% and ~ 54% lower risk for reinfection and reinfection-associated severe COVID-19, respectively, modified by time-elapsed since the last immunity-generating event, when compared against complete primary schedules.

**Conclusion:**

SARS-CoV-2 reinfections increased during Omicron predominance. Hybrid immunity provides protection against reinfection and associated severe COVID-19, with potential benefit from heterologous booster schedules.

## Introduction

As the COVID-19 pandemic continues, a larger number of previously infected individuals have become reinfected with SARS-CoV-2 (1). Evidence of SARS-CoV-2 reinfection was first documented in August 2020 (2) and was initially considered a rare event (1, 3–7). Omicron SARS-CoV-2 variant and its subvariants have been shown to possess a higher capacity for immune escape (8, 9) and transmissibility (10), which resulted in the most significant increase of infection and reinfection rates as it became the dominant variant in circulation (11). With Omicron, approximately 41% of some countries’ population are estimated to be at risk of SARS-CoV-2 reinfection (12). Although SARS-CoV-2 reinfections are described as less severe than primary infections (13), severe events continue to be reported despite increasing vaccination rates (3, 14); furthermore, some studies have reported no difference in severity between prior infections and reinfections (15, 16). For individuals who survive reinfections, all-cause mortality and hospitalization risk in the acute and post-acute phase has been reported, as well as a relationship between the frequency of COVID-19 reinfections and the prevalence of post-acute COVID-19 conditions (17).

Individuals with previous infection and at least one dose of a COVID-19 vaccine are benefitted from hybrid immunity, where natural immunity due to infection and vaccination-acquired immunity interact to enhance protection against reinfection and severe disease. As evidence supporting the protective role of hybrid immunity over either natural or vaccine-acquired immunity alone continues to arise (18–21), the complexity of this phenomenon becomes more evident. A series of different factors, such as the number of vaccine doses received, vaccine platform used, the severity of the first COVID-19 episode, SARS-CoV-2 variants, and subvariants responsible for both first infection and reinfection and time-dependent waning protection seem to have a significant effect on the level of protection conferred by hybrid immunity (22–28). Furthermore, since immune imprinting, a phenomenon in which B-cell immune response from first exposure to antigens related to an infectious agent (either through vaccination or previous infection) was demonstrated to condition the host response toward SARS-CoV-2 reinfections (10, 29, 30), studies have started to include the order in which immunity-generating events (vaccination and infection) occur as an additional factor to be considered in order to understand hybrid immunity better (31–33). Even though real-world evidence of hybrid-immunity protection continues to emerge, studies integrating the order of immunity-generating events are still scarce. Hybrid-immunity evidence is still missing in Latin American countries, which, due to their social determinants of health, continue to experience the COVID-19 pandemic differently from high-income countries (34). Here, we evaluated the role of primary infection and hybrid immunity on the risk of reinfection during periods of Omicron variant predominance in Mexico in individuals with a previous SARS-CoV-2 infection. We also evaluated the influence of the order of immunity-generating events, previous hospitalization, and SARS-CoV-2 variant predominance on the risk of reinfection and reinfection-associated severe outcomes using a nationwide COVID-19 registry.

## Methods

### Study population

**Figure 1:**
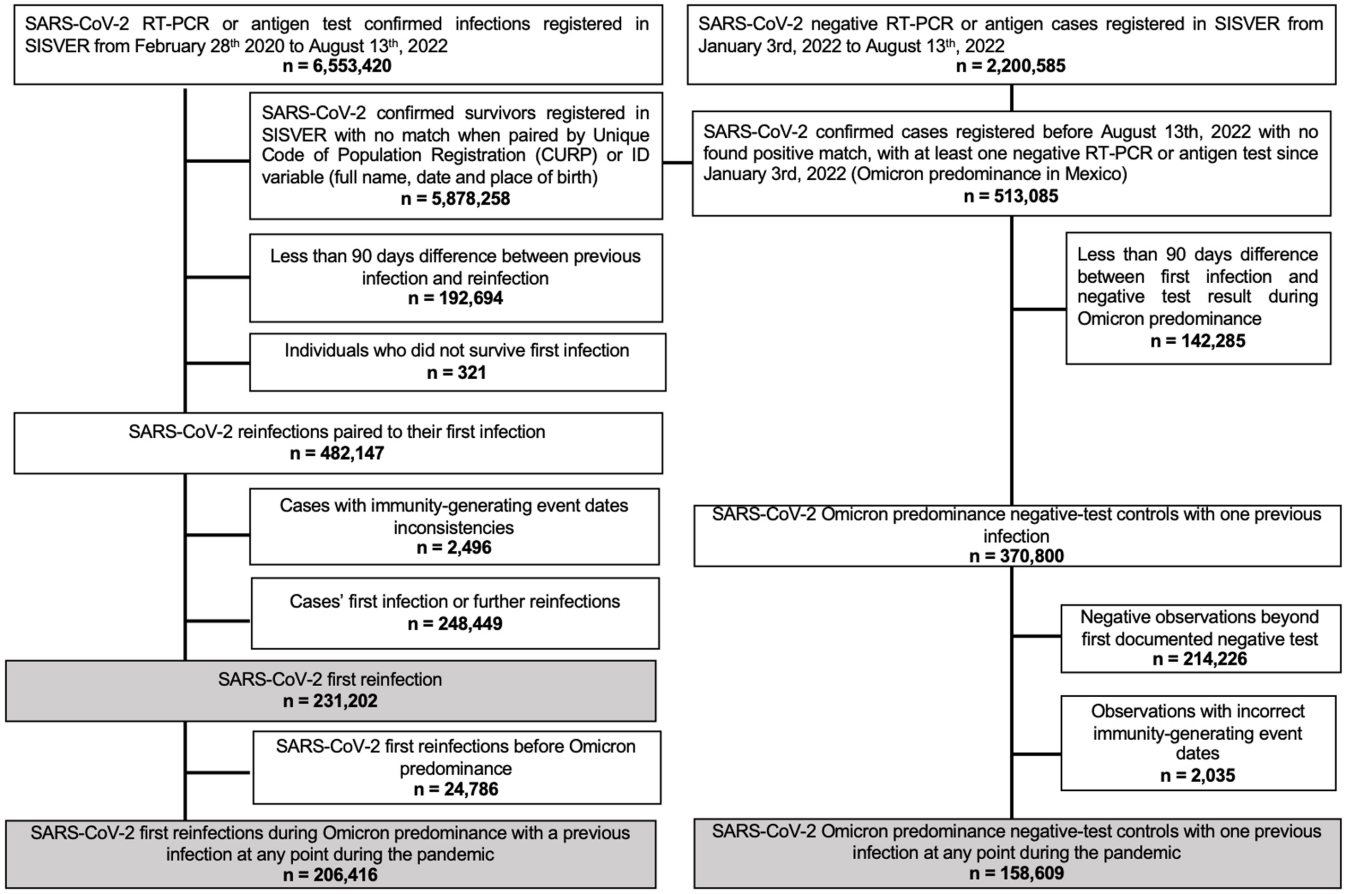
Flowchart of included and excluded subjects with SARS-CoV-2 documented reinfection or negative test as reported in SISVER from March 3rd, 2020, to August 13th, 2022.

We assessed cases of suspected SARS-CoV-2 reinfection using the SISVER registry, a daily updated nationwide surveillance system of suspected SARS-CoV-2 cases in Mexico (35, 36), managed by the General Directorate of Epidemiology of the Mexican Ministry of Health. Detailed sociodemographic and clinical information is ascertained, including details of SARS-CoV-2 infection and clinical course, as well as vaccination status, date, and vaccine applied. For this analysis, from March 3rd, 2020, to August 13th, 2022, we analyzed survivors of a first confirmed SARS-CoV-2 infection with suspected reinfection during the predominance of Omicron. Only suspected reinfections which were separated at least 90 days from primary infection and with either a positive or negative SARS-CoV-2 RT-PCR or antigen test were included. A flowchart of included and excluded subjects is presented in Figure 1.

### Definition of immunity-generating events

An immunity-generating event was considered either a vaccination against COVID-19 (first, second dose, or booster shot) or a confirmed SARS-CoV-2 infection, either primary or reinfection. Reinfections were defined as a confirmed SARS-CoV-2 infection using RT-PCR or antigen test at least 90 days after confirmed primary SARS-CoV-2 infection (37). Nationally-available SARS-CoV-2 vaccines applied in Mexico during this period included BNT162b2, mRNA-1,273, Gam-COVID-Vac, Ad5-nCoV, Ad26.COV2.S, ChAdOx1-S, NVX-CoV2373, and CoronaVac (38). The vaccination schedule for most vaccines considered two doses, except for one-dose Ad5-nCoV and Ad26.COV2.S vaccines. Individuals were considered fully vaccinated if they had completed the vaccination schedule ≥14 days before the evaluated outcome (39, 40). Partially vaccinated individuals were considered if they had one out of a two-dose vaccine schedule or if the outcome happened <14 days in an otherwise completely vaccinated individual. COVID-19 vaccines were categorized according to their platform. Booster vaccination was considered if fully vaccinated individuals received an additional dose of a COVID-19 vaccine and at least 7 days had elapsed since vaccination (41), otherwise were reclassified as fully vaccinated. Booster schedules were categorized as homologous if booster shots were the same as the primary vaccination and heterologous if different.

#### Determinants of reinfection and severe COVID-19 risk

Previous evidence has shown varying degrees of protection for SARS-CoV-2 reinfection and severe COVID-19 (defined by hospitalization, ICU admission, intubation, or death) based on the variant responsible for primary and second infection (42, 43), time since vaccination or primary infection (24, 43), and antibody response (44–46). Therefore, we evaluated the following variables:

1. *Predominant SARS-CoV-2 variant for first infection* – A fraction of COVID-19 samples are sequenced by authorized national laboratories and submitted to the GISAID platform. Infection was assumed to be most likely caused by the predominant variant based on the date of symptom onset. Based on data submitted to GISAID (47), from March 3rd, 2020, until March 30th, 2021, the predominant SARS-CoV-2 variant was the ancestral strain, followed by the predominance of the B.1.1.519 variant until June 6th, 2021, the P.1 (Gamma) variant until July 4th, 2021, the B.1.617.2 (Delta) variant until January 2nd, 2022. B.1.1.529.1 (Omicron) BA.1 subvariant was considered from January 3rd, 2022, to April 24th, 2002, followed by Omicron subvariant BA.2 until June 19th, 2022.
2. *Predominant SARS-CoV-2 variant for reinfection* – Categorized based on the date of symptom onset for the reinfections, according to GISAID. Periods of Omicron subvariants BA.1 and BA.2 dominance were the same as previously described. Predominance of the BA.4 subvariant was considered from June 20th to July 3rd, 2022, followed by BA.5 subvariant predominance until August 13th, 2022.
3. *Previous COVID-19-related hospitalization* – Describes an individual who had been hospitalized and survived their primary SARS-CoV-2 infection.
4. *Order of immunity-generating events* – We developed an indicator variable that considered the order of immunity-generating events for a given individual, using as reference unvaccinated individuals with primary SARS-CoV-2 infection (Supplementary material). This variable considered the order of first infection, partial, complete, or booster vaccination before their second SARS-CoV-2 testing for suspected reinfection.
5. *Time elapsed since last immunity-generating events* – Defined as time elapsed in months since the last immunity-generating event, either vaccination or infection. Time was categorized based on whether individuals had ≥6 months since exposure, given previous evidence of vaccination or previous infection immunity waning over this period (48).

### Statistical analyses

#### Epidemiology of SARS-CoV-2 reinfections in Mexico

We characterized all SARS-CoV-2 reinfections in Mexico to explore sociodemographic and clinical characteristics. Reinfection incidence and mortality were calculated over the number of individuals who survived a confirmed primary SARS-CoV-2 infection. The number of confirmed reinfections was plotted over time and based on occurrence during periods of variant predominance in Mexico for the first and second SARS-CoV-2 infections using cross-tabulation matrix plots to visualize combinations of reinfections (Supplementary material).

#### Determinants of reinfection and reinfection-associated severe COVID-19 risk

We matched SARS-CoV-2 reinfections to individuals with a negative SARS-CoV-2 test after primary infection using propensity score matching for age and sex. Next, we fitted conditional logistic regression models, including matching weights to explore the role of previously defined determinants on the risk of reinfection. We also explored interaction effects between the order of immunity-generating events and time elapsed since last exposure to an immunity-generating or hospitalization during primary SARS-CoV-2 infection. For models on the risk of severe COVID-19 associated with reinfection, we only analyzed cases with confirmed reinfection and explored factors as described earlier.

#### Heterologous versus homologous vaccine boosting

Given the diversity of COVID-19 vaccination schedules applied in Mexico, we explored the risk of reinfection and severe COVID-19 associated with homologous and heterologous booster protocols compared to fully vaccinated individuals, exploring similar determinants as described above using conditional logistic regression.

## Results

### Epidemiology of SARS-CoV-2 reinfections in Mexico

**Figure 2:**
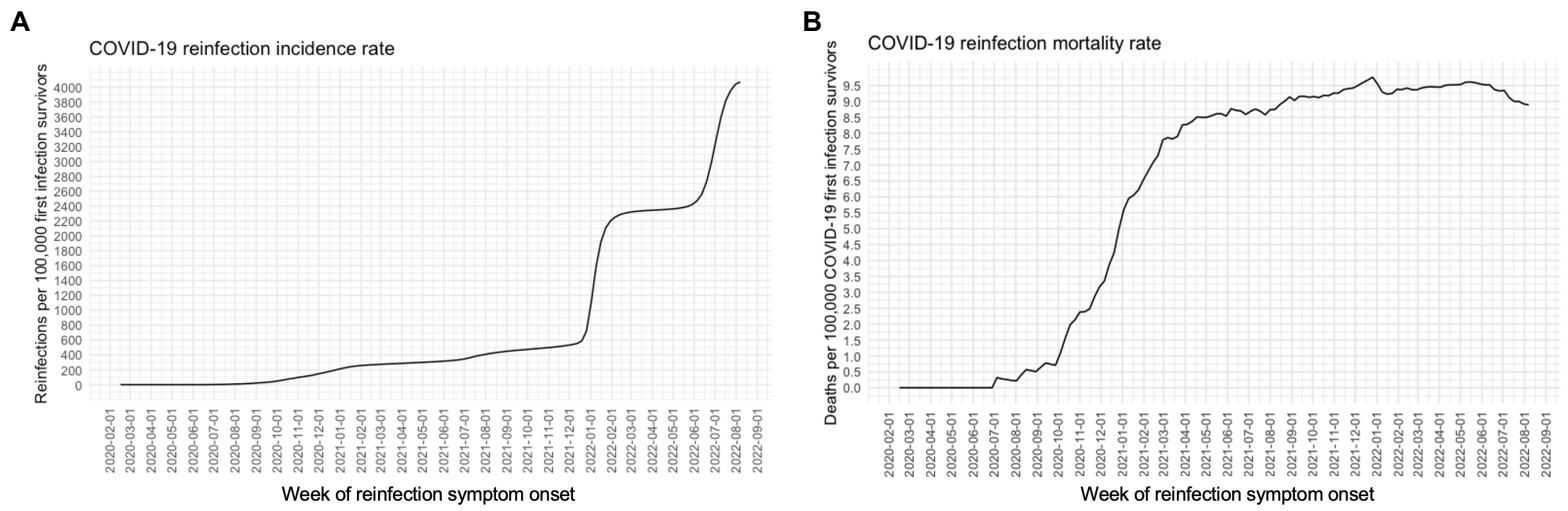
Incidence rates of SARS-CoV-2 cases associated with reinfections **(A)** and reinfection-associated deaths **(B)** per 100,000 first SARS-CoV-2 infection survivors in Mexico from March 3rd, 2020, until August 13th, 2022 (*n* = 231,202).

We detected 231,202 confirmed reinfections over the study period, with most reinfections occurring during January and June 2022. A steady rate of reinfection-associated mortality was observed starting from March 2021 (Figure 2). Most SARS-CoV-2 reinfections occurred in women (60.12%) and individuals aged 31–40 years [30.26% and a median time between reinfections of 362 days (IQR 196–531 days); Supplementary material]. Reinfections occurred primarily in unvaccinated individuals (41.55%) or cases in which primary infection occurred before completing vaccination schedules or receiving a booster shot (41.0%). Reinfections in individuals with comorbid diabetes or obesity were low (5.97 and 8.34%). Most primary infections occurred during the predominance of ancestral strains (50.06%), followed by Delta (23.1%) and Omicron BA.1 (14.67%). Over 206,416 reinfections occurred during periods of predominance of the Omicron variant in Mexico (89.3%), primarily associated with the predominance of Omicron BA.1 (36.74%) and Omicron BA.5 (33.67%) subvariants. We identified 3,261 hospitalizations related to reinfections (1.41%), and 515 deaths, with a reinfection fatality rate of 0.22% (Supplementary Table S1). Overall, we identified a peak of reinfections with a stable mortality rate was observed since the start of Omicron predominance, mainly affecting incompletely or not vaccinated individuals.

### Risk of SARS-CoV-2 reinfection with hybrid immunity

**Figure 3:**
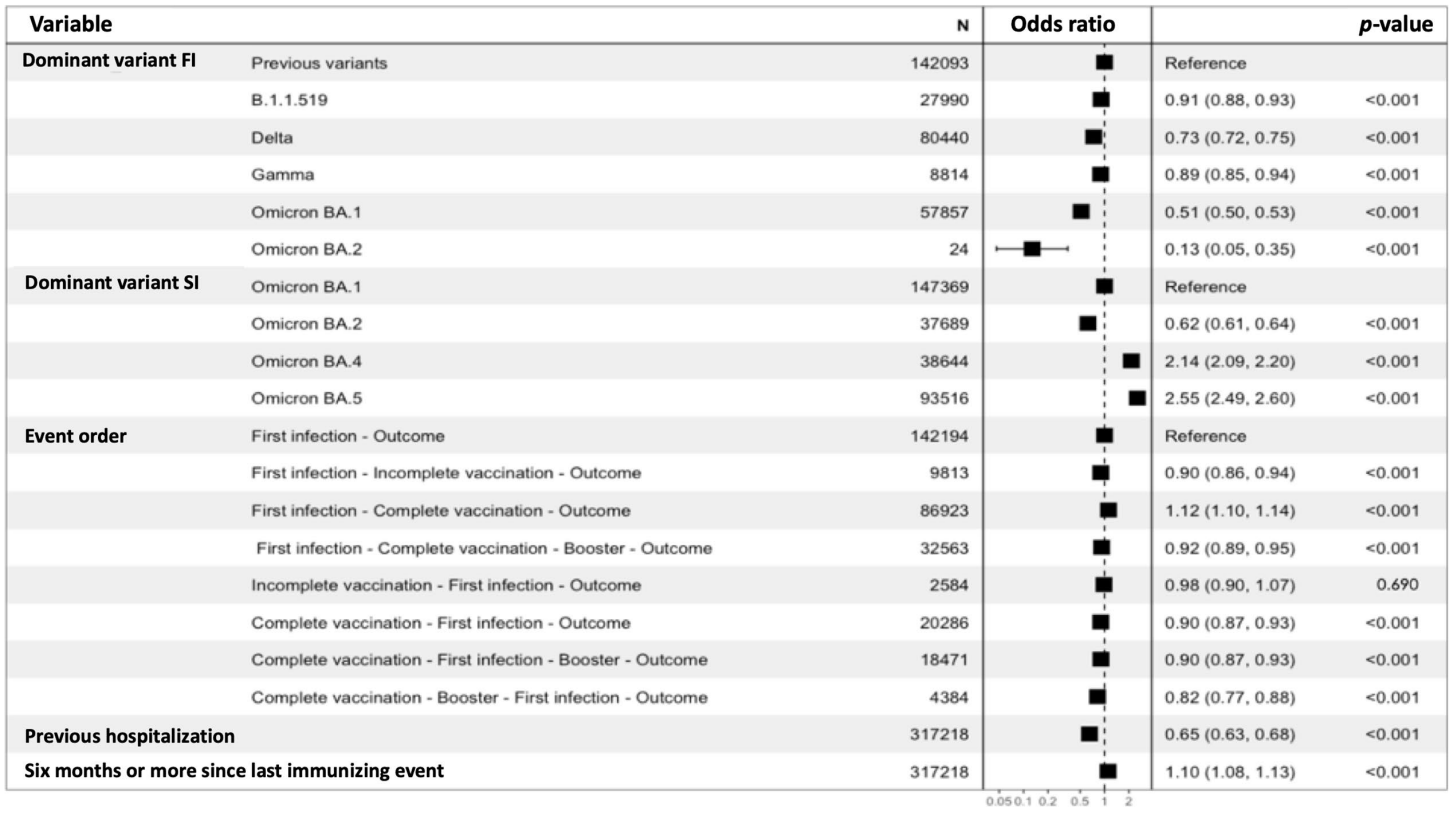
Conditional logistic regression model for risk of reinfection in subjects with confirmed reinfection (*n* = 158,609) paired with subjects with first confirmed SARS-CoV-2 reinfection and a second negative test taken at least 90 days after the first infection (*n* = 158,609). Pairing was performed using propensity score matching for age and sex. FI, first infection; SI, second infection.

We paired 158,609 cases of confirmed reinfection with 158,609 controls with primary infection and a second negative SARS-CoV-2 test taken at least 90 days after primary infection (Supplementary material). Decreased risk of reinfection was associated with a primary infection during the predominance of most SARS-CoV-2 variants compared to the ancestral strain, with higher protection observed for the predominance of Gamma, Omicron BA.1, and BA.2 subvariants (Figure 3). Increased risk of reinfection was associated with the predominance of the Omicron BA.4 and BA.5 subvariants, compared to periods of BA.1 subvariant predominance. Hospitalization during the first SARS-CoV-2 infection was associated with a lower risk of reinfection, while ≥6 months since the last immunity-generating event was associated with a higher risk. The order of immunity-generating events did not significantly impact the risk of reinfection compared to unvaccinated individuals with primary infection; however, the lowest risk was observed in fully boosted individuals prior to primary infection. A paradoxical increase in the risk of reinfection was observed in subjects fully vaccinated after primary infection; nevertheless, a higher risk in this category was observed for subjects with ≥6 months since the last immunity-generating event. When stratifying models according to variant predominance at the time of reinfection, no significant changes were observed for these associations (Supplementary material). Primary infection during the predominance of Omicron protected the best against reinfection by this same SARS-CoV-2 variant, with slight variation between subvariants and hybrid-immunity profiles.

### Risk of severe COVID-19 associated with reinfections

**Figure 4:**
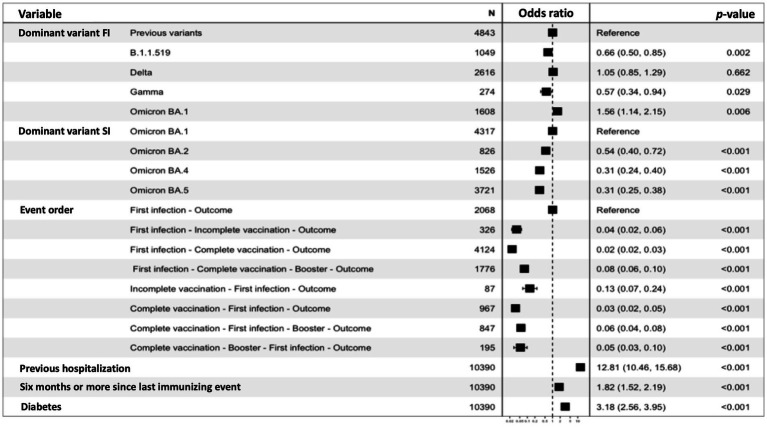
Conditional logistic regression model for risk of severe COVID-19, defined as an event of hospitalization, the requirement for ICU admission, intubation, or death in subjects with confirmed SARS-CoV-2 reinfection and severe COVID-19 (*n* = 2,078) compared to mild SARS-CoV-2 reinfections (*n* = 8,312), paired using propensity score matching for age and sex. FI, first infection; SI, second infection.

We explored risk factors in subjects with confirmed reinfection and severe COVID-19 (*n* = 2,078) paired 1:4 with subjects with reinfection without severe COVID-19 (*n* = 8,312) using propensity score matching for age and sex. Regarding primary infection, a decreased risk for severe COVID-19 was observed during the period of Gamma predominance compared to that of the ancestral strain. An increased risk was associated with primary infection during Omicron BA.1 subvariant predominance. A progressively decreased risk of severe COVID-19 was observed during periods of Omicron BA.2, BA.4, and BA.5 predominance, compared to reinfection during the predominance of the BA.1 subvariant (Figure 4). Compared to unvaccinated individuals, all combinations of immunity-generating events that included at least one vaccine dose were associated with a > 90% reduction in the risk of severe COVID-19, except for incomplete vaccination prior to primary infection, providing a ~ 87% reduction in risk. Having been hospitalized and survived the primary SARS-CoV-2 infection was associated with a higher risk of severe COVID-19 during reinfection, as well as having ≥6 months or more since the last immunity-generating event and having comorbid diabetes. Additional adjustment for age and sex to control for residual confounding did not modify these associations. Vaccination yielded the most considerable reduction in the risk of severe COVID-19 associated with reinfection, while previous hospitalization due to COVID-19, followed by diabetes, was related to a higher risk.

### Homologous versus heterologous boosters and risk of reinfection and severe outcomes

**Figure 5:**
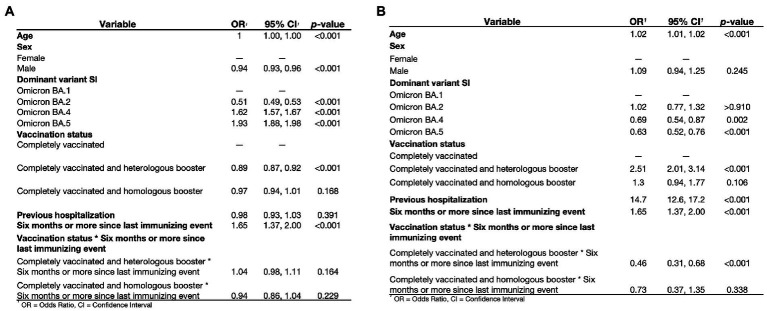
Conditional logistic regression model for risk of reinfection **(A)** and severe COVID-19 **(B)** in subjects with complete vaccination protocol (*n* = 121,727) with or without an additional booster shot (*n* = 62,259) to evaluate the impact of heterologous or homologous boosting in subjects with first confirmed SARS-CoV-2 infection. SI, second infection.

The most frequently reported booster shot in subjects evaluated for reinfection was heterologous vaccination of BNT162b2 boosted with ChAdOx1-S (40.6%), followed by homologous vaccination with ChAdOx1-S (23.5%), homologous vaccination with BNT162b2 (5.3%), and heterologous vaccination of Ad5-nCoV boosted with mRNA-1,273 (4.8%, Supplementary material). We observed 102,634 reinfections in fully vaccinated or boosted individuals with primary infection (*n* = 183,986, 55.8%), among which 67,880 occurred in fully vaccinated individuals without boosting (*n* = 121,727, 55.8%) and 34,754 occurred in individuals with boosters (*n* = 62,259, 55.8%). Risk factors for SARS-CoV-2 reinfection included older age and female sex, while a higher risk was observed for reinfections in periods of Omicron BA.4 and BA.5 predominance (Figure 5A). When compared against fully vaccinated individuals without boosting, heterologous boosters were associated with ~11% decreased risk of reinfection, with no significant difference from homologous boosters. Cases with ≥6 months since the last immunity-generating event had a higher risk of reinfection. Increased risk of reinfection-associated severe COVID-19 was observed for older age while infection during the predominance of Omicron BA.4 and BA.5 subvariants was associated with decreased risk (Figure 5B). Subjects with heterologous boosters and the last immunity-generating event having occurred at least 6 months before had ~54% lower risk of severe COVID-19 than completely vaccinated individuals with ≥6 months from exposure. Notably, no differences were observed for homologous boosters compared to subjects without boosting. Heterologous booster schedules were associated with a lower risk of both reinfection and severe reinfection even after 6 months or more from the last immunizing event, compared to complete primary and homologous booster schedules.

## Discussion

Here, we conducted one of the largest evaluations of the risk of reinfection and severe COVID-19 by analyzing 231,202 individuals with primary SARS-CoV-2 infection in Mexico, with 89.3% reinfections occurring during the period of Omicron predominance. In this setting, we evaluated the role of hybrid immunity, the severity of primary infection, and the influence of time-waning immunity in the risk of reinfection and reinfection-related severe COVID-19 risk. As previously reported, most reinfections were associated with Omicron and its subvariants (28, 31, 49, 50), rapidly accelerating reinfection rates during BA.1 and BA.5 subvariant predominance and an intermediate plateau attributed to a stable primary infection-reinfection ratio. The predominance of Omicron subvariants did not modify reinfection-associated death rates. Vaccination protected against reinfection without significant influence from the order of immunity-generating events; nevertheless, the highest degree of protection was observed for fully vaccinated individuals boosted prior to their primary infection suggesting that, in fully vaccinated and boosted naïve individuals, primary infection is an effective additional immune booster against reinfection (51). Similar to previously reported evidence of antibody response to primary schedule vaccination with mRNA vaccines, after primary infection, one out of a two-dose SARS-CoV-2 vaccine schedule seemed to provide similar protection against reinfection as a complete two-dose schedule (33). Likewise, a complete schedule before primary infection would provide better protection against reinfection than an incomplete schedule, as previously suggested by serological studies for BNT162b2 mRNA vaccine (32). However, the protection provided by the different vaccine-primary infection profiles only differed modestly.

As for severe COVID-19 reinfections, primary infection with B.1.1.519 (52) or the Gamma variant yielded the highest protection among other variants and subvariants. Omicron BA.1 primary infection was related to a higher risk. Hospitalization during primary infection, 6 months or more since the last immunity-generating event, and comorbid diabetes conferred a higher risk of severe COVID-19 reinfection. Finally, we observed the superiority of heterologous boosters over complete vaccine schedules for protection against reinfection or severe COVID-19, confirming previous reports on the benefits of heterologous vaccination against COVID-19 (53, 54).

Considering that approximately ~50% of Mexican adults have antibodies against the Nucleocapsid protein of SARS-CoV-2, indicating exposure and likely a previous infection (55), and given the widespread circulation of Omicron, seroprevalence most likely increased along with the proportion of the population at risk of reinfection (56). The increased susceptibility to reinfection was demonstrated by the growing trends in the proportion of reinfections found in this study, representing as much as 12% of the weekly total COVID-19 cases (Supplementary material), which, interestingly, is a similar proportion to that reported in a Serbian study on reinfections after the advent of the Omicron variant (57). However, this proportion is likely higher due to limitations of case definitions and reporting. This proportion is expected to continue growing following the appearance of new variants. The contribution of COVID-19 vaccines to hybrid immunity wanes within a few months of vaccination, particularly in the context of each new variant of concern (58); however, effectiveness is still maintained for Omicron and its subvariants, particularly against severe COVID-19. Therefore, vaccines are observed to meet the strategic objectives highlighted in the WHO’s SAGE Roadmap for prioritizing the uses of COVID-19 vaccines by preventing severe reinfections (59).

Among the strengths of our study, we highlight that it represents one of the largest reports of SARS-CoV-2 reinfections and risk factors in a country with high SARS-CoV-2 seroprevalence and broad vaccination coverage. Furthermore, given the diversity of the epidemiological situation in Mexico throughout each of the 32 states and the variety of vaccines employed, the use of an epidemiological surveillance dataset that concentrates information from a national level of not only confirmed cases but all suspected SARS-CoV-2 infections along with their laboratory test results allows for adequate assessment of reinfections and their associated outcomes, as well as the order of immunity-generating events, making it possible to address the heterogeneity and complexity of hybrid immunity. Finally, given the diversity of vaccines used in Mexico, we could also provide real-world evidence on the effectiveness of combinations of incomplete, complete, and booster vaccine schedules with primary infection and heterologous and homologous vaccine boosters in subjects with prior SARS-CoV-2 infection. Among the limitations to be acknowledged is the definition of reinfection. Recent evidence demonstrates that reinfections can occur within a shorter period and may incorrectly exclude some reinfections in our study (60).

Furthermore, when analyzing risk associated with SARS-CoV-2 variants, we assumed that predominant variants likely caused infections during each period; nevertheless, this type of inference has been used as an approach to variant analysis when individual-level genomic data is unavailable (10, 31, 48, 52). This approach limits our capacity to differentiate the effect of high community transmission pressure on reinfections from the ability of variants to evade hybrid immunity. However, the higher transmissibility of new SARS-CoV-2 variants is intimately related to their virulence and should therefore be considered a consequence of the latter. Finally, we did not perform individual analyses for each vaccine and booster combination, provided that not all combinations had a large enough number of outcomes to allow for adequate comparisons. The role of vaccination and boosters’ interaction with predominant circulating variants remains an area of opportunity for future research. Given that most reinfections occurred during periods of Omicron predominance and that immunity-boosting by infection with Omicron seems to be low, real-world studies on hybrid immunity that consider the effects of immune imprinting from SARS-CoV-2 on protection against reinfection will be fundamental for determining the need of booster shots and its frequency in future vaccination waves.

Even though protection against reinfection conferred by boosters appears to decrease following the advent of new variants, protection against severe reinfection remains high. Hybrid-immunity studies should place the focus on severe disease and individuals prone to it—specifically, people at high risk for serious illness, older adults, individuals at high risk of exposure (61), immunocompromised individuals, and comorbid conditions (62) such as diabetes and hospitalization during first SARS-CoV-2 infection, both for which an association with severe reinfection was found in our study.

As of now, vaccination continues to be our most robust defense against COVID-19, regardless of previous exposure to SARS-CoV-2. Primary and booster vaccination should therefore be prioritized in those unvaccinated or those who have not received their first booster shot, providing additional protection against reinfection (31) and lowering the risk of post-acute COVID-19 syndrome associated with reinfection; but mainly preventing severe outcomes, particularly for variants with increased transmission and associated immune evasion such as Omicron (63). As reinfections become more frequent (64), surveillance systems may benefit from the study of SARS-CoV-2 reinfections.

## Research in context

### Evidence before this study

We searched PubMed for the terms “SARS-CoV-2” AND “reinfection” AND “hybrid immunity” until November 20th, 2022, and identified a few population studies previously conducted in Israel, Sweden, Qatar, United States, and Canada which explored the risk of reinfection and the protective role of hybrid immunity in individuals with one, two, or three doses of COVID-19 vaccines, predominantly during periods of the predominance of Omicron BA.1 and BA.2 subvariants. Notably, no studies were conducted in any Latin American country or reported on the benefit of heterologous booster schemes or the order of immunity-generating events.

### Added value of this study

We report the results of a nationwide study in Mexico of over 230,000 SARS-CoV-2 reinfections, with ~90% occurring during periods of Omicron predominance. We identified that vaccination provided additional benefits in reducing the risk of SARS-CoV-2 reinfection, with the highest benefit observed in individuals with complete vaccination and booster protocols prior to primary infection or with primary infection during periods of BA.1 and BA.2 subvariant predominance. Hybrid immunity also substantially reduces the risk of reinfection-associated severe COVID-19, with a > 90% reduction in risk compared to unvaccinated individuals with previous SARS-CoV-2 infection, regardless of the order of immunity-generating events. Finally, heterologous COVID-19 booster schedules were associated with ~11% and ~ 54% lower risk for reinfection and reinfection-associated severe COVID-19, respectively, modified by time-elapsed since the last immunity-generating event, when compared against complete primary schedules.

### Implications of all the available evidence

Our results support that COVID-19 vaccination and boosters provide additional benefits to protect against SARS-CoV-2 reinfection and reinfection-associated severe COVID-19. Using heterologous boosters appears to provide additional protection in previously infected individuals. Such schemes may prove beneficial as newer and more transmissible variants emerge.

## Data availability statement

The datasets presented in this article are not readily available because of privacy restrictions. Requests to access the datasets should be directed to the General Directorate of Epidemiology of Mexico (DGE).

## Ethics statement

The studies involving human participants were reviewed and approved by the Ethics and Research Committee at Instituto Nacional de Geriatría, project number DI-PI-005/2021. The ethics committee waived the requirement of written informed consent for participation.

## Author contributions

JM-G and OB-C: research idea and study design and statistical analysis. JM-G, CZ-J, RG-V, GG-R, and HL-G: data acquisition. JM-G, OB-C, NA-V, CF-M, DR-G, and AV-V: data analysis/interpretation. JM-G, OB-C, NA-V, CF-M, DR-G, AV-V, and SV-F: manuscript drafting. OB-C: supervision or mentorship. All authors contributed important intellectual content during manuscript drafting or revision and accepted accountability for the overall work by ensuring that questions pertaining to the accuracy or integrity of any portion of the work are appropriately investigated and resolved.

## Funding

This research was supported by Instituto Nacional de Geriatría.

## Conflict of interest

The authors declare that the research was conducted in the absence of any commercial or financial relationships that could be construed as a potential conflict of interest.

## Publisher’s note

All claims expressed in this article are solely those of the authors and do not necessarily represent those of their affiliated organizations, or those of the publisher, the editors and the reviewers. Any product that may be evaluated in this article, or claim that may be made by its manufacturer, is not guaranteed or endorsed by the publisher.
